# Effectiveness and Tolerability of an Emollient “Plus” Compared to Urea 10% in Patients With Mild‐to‐Moderate Atopic Dermatitis

**DOI:** 10.1111/jocd.70051

**Published:** 2025-02-19

**Authors:** Cita Rosita Sigit Prakoeswa, Berliana Kurniawati Nur Huda, Ditya Indrawati, Menul Ayu Umborowati, Sylvia Anggraeni, Damayanti, Dwi Murtiastutik, Delphine Kerob

**Affiliations:** ^1^ Department of Dermatology, Venereology and Aesthetic Dr. Soetomo General Academic Hospital Surabaya Indonesia; ^2^ Department of Dermatology, Venereology and Aesthetic Faculty of Medicine Universitas Airlangga Surabaya Indonesia; ^3^ La Roche‐Posay Laboratoire Dermatologique Levallois Perret France

**Keywords:** atopic dermatitis, emollient “plus”, randomized controlled trial, skin barrier function, urea 10% moisturizer

## Abstract

**Background:**

Atopic dermatitis (AD) poses a challenge due to its chronic inflammatory nature. Recent research highlights microbiome dysbiosis as a key contributor. Emollients “plus” are modern moisturizers containing bacterial lysate, improving skin barrier function and reducing *Staphylococcus aureus* colonization, thus mitigating AD symptoms. Emollient “plus” containing *Vitreoscilla filiformis* biomass (*Aqua Posae filiformis*) is efficient in AD, as single adjunct for milder forms or adjunctive to systemic treatments in more severe forms. Standard recommended moisturizers for AD in Indonesia contain urea 10%.

**Aims:**

This trial compared an emollient “plus” (Group A) with urea 10% moisturizer (Group B) in the treatment of mild‐to‐moderate AD.

**Patients/Methods:**

Sixty subjects with mild‐to‐moderate AD were randomized into Groups A and B (30 subjects/group). Test products were applied twice daily for 12 weeks. Clinical and instrumental endpoints assessed at Weeks 0, 4, 8, and 12 included Severity Scoring of AD (SCORAD), Pruritus Visual Analog Scale (PVAS), Eczema Area and Severity Index (EASI), Dermatology Life Quality Index (DLQI), Transepidermal Water Loss (TEWL), skin hydration, skin pH, as well as tolerance evaluation.

**Results:**

Significant differences in favor of the emollient “plus” versus urea 10% were observed on TEWL and skin pH values at Weeks 4, 8, and 12, on SCORAD and skin hydration values at Weeks 8 and 12. EASI, DLQI, and PVAS values differed significantly at Week 12 in favor of Group A. Both products were well tolerated.

**Conclusions:**

This emollient “plus” has superior efficacy in improving AD symptoms and skin barrier function compared to urea 10% moisturizer.

## 1 Introduction

Atopic dermatitis (AD) is a chronic inflammatory skin disease that is associated with itching and pain and has the potential to disrupt individual's quality of life. One of the pathogenesis of AD that has been widely studied recently is microbiome dysbiosis which also contributes to worsening the symptoms of AD itself [1]. Of note, cutaneous dysbiosis may amplify barrier dysfunction in patients with AD and early‐onset barrier dysfunction may facilitate an innate immune response to commensal organisms and, consequently, the development of allergic sensitization [2]. In addition, pathogens in the skin, such as *Staphylococcus aureus* ( *S. aureus* ), can induce Interleukin (IL)‐9 in AD. Interleukin (IL)‐9 is involved in Th2‐mediated allergic inflammation and plays a pleiotropic role in allergic disease by promoting eosinophil and mast cell infiltration, also IgE secretion which impacts on AD severity [3]. Research in recent decades has explored how topical pre‐, post‐, and probiotic therapies can improve AD while rebalancing a diverse skin microbiome. Daily use of emollients is recommended by AD guidelines to restore skin barrier, prevent flares, and further decrease the need of topical steroids to overcome their known side effects [4, 5]. Emollients “plus” are recommended in European guidelines for AD, as they contain active ingredients that can balance the microbiome in AD skin patients [6]. In these guidelines, emollients are defined as “topical formulations with vehicle‐type substances lacking active ingredients,” whereas emollients “plus” refers to “topical formulations with vehicle‐type substances and additional active, non‐medicated substances.” These active ingredients may contain, for example, saponins, flavonoids, and riboflavins from protein‐free oat plantlet extracts or bacterial lysates from *Aquaphilus dolomiae* or *Vitreoscilla filiformis* .

One of the active ingredients mentioned in the guidelines is bacterial lysate produced from *Vitreoscilla filiformis* (Vf). *Vitreoscilla filiformis* biomass has been shown to have antioxidant and antibacterial properties and also has the ability to stimulate innate skin defense [7]. The Vf lysate grown in thermal spring water is named *Aqua Posae filiformis* (APF). Maintaining a healthy physical and microbial skin barrier integrity is important in the management of AD, especially preventing the overproliferation of *S. aureus* which contributes to flares and worsening of AD [1]. Recently, emollients “plus” have become popular as the next generation of emollients, with the explicit goal of improving AD lesions by rebalancing the skin microbiome. They have demonstrated their efficacy either in monotherapy or in adjunct to topical or systemic drugs in the management of AD, improving clinical signs and symptoms of AD, reducing the need of topical steroids, while rebalancing the skin microbiota, making it the most cost‐effective method to preventing AD recurrence [8]. The balm used in the present study, referred to as “emollient ‘plus’” hereafter, contains key ingredients such as shea butter, canola oil, niacinamide, APF, and microresyl to strengthen skin physical barrier, rebalance skin microbiome by avoiding overgrowth of *S. aureus* and its biofilm formation, and decrease inflammation.

In the Allergy Division Dermatology and Venereology outpatient clinics of Dr. Soetomo, General Academic Hospital Surabaya, urea 10% moisturizer and topical corticosteroids are the standard of topical therapy for AD. This study aimed to compare the effectiveness and tolerability in patients with mild‐to‐moderate AD of an emollient “plus” to urea 10% moisturizer through clinical improvement parameters including Severity Scoring of AD (SCORAD), Pruritus Visual Analog Scale (PVAS), Eczema Area and Severity Index (EASI), Dermatology Life Quality Index (DLQI), and instrumental evaluations including Transepidermal Water Loss (TEWL), skin hydration, skin pH, as well as tolerance evaluation.

## 2 Materials and Methods

This double‐blind, randomized, controlled clinical trial was designed to compare the clinical effectiveness and tolerability of an emollient “plus” (Group A, Lipikar AP+M balm, La Roche‐Posay Laboratoire Dermatologique) with urea 10% moisturizer (Group B) in subjects with mild‐to‐moderate AD. A total of 60 subjects were to be randomized into 2 groups. Both moisturizers were used twice daily for 12 weeks, along with standard topical steroid therapy of the lesions to manage flares of AD [9]. Clinical parameters (SCORAD, PVAS, EASI, and DLQI) and instrumental parameters (TEWL, skin hydration, and skin pH) were evaluated at Week 0 (Baseline) and Weeks 4, 8, and 12. Side effects induced by the moisturizers were also recorded during the study.

Randomization was done by simple random sampling using numbers table. The process ensures that every subject has an equal opportunity to be allocated to Group A or Group B.

Both standard and emollient “plus” were repackaged similarly by third parties and labeled using codes “A” and “B.” Thus, neither researchers nor subjects were aware of the substance given (double blinded). The codes were revealed when the study was over.

The patients in this manuscript have given written informed consent to publication of their case details including photographs.

Statistical analyses employed for within‐group comparisons were using the Wilcoxon Signed Ranks test. To examine the inter‐group comparisons, the Mann–Whitney test was performed. The Friedman test, a non‐parametric statistical test, was used to analyze differences between the categorical data.

## 3 Results

A total of 60 subjects were enrolled in this study, 30 subjects being randomized in each treatment group. Baseline demographic and disease characteristics were comparable between the groups (Table 1), notably in terms of severity of AD with 15 subjects having mild and 15 subjects having moderate AD in each group.

**TABLE 1:** Baseline demographic and disease characteristics.

	Group A: emollient “plus”	Group B: urea 10%
Gender, *n* (%)		
Male	5 (16.7)	7 (23.3)
Female	25 (83.3)	23 (76.7)
Average age (years)	36.77	38.10
AD severity based on SCORAD, *n* (%)		
Mild	15 (50)	15 (50)
Moderate	15 (50)	15 (50)
Skin pH, mean (SD)	6.35 (0.54)	6.50 (0.57)
Skin hydration, mean (SD)	2.24 (3.59)	1.49 (2.80)
SCORAD, mean (SD)	25.55 (8.95)	25.34 (7.27)
EASI, mean (SD)	2.40 (2.27)	2.25 (2.22)
DLQI, mean (SD)	8.13 (4.75)	10.07 (5.06)
TEWL, mean (SD)	14.41 (4.70)	15.91 (5.92)
PVAS, mean (SD)	5.97 (1.52)	5.40 (1.50)

Abbreviation: SD, standard deviation.

**FIGURE 1:**
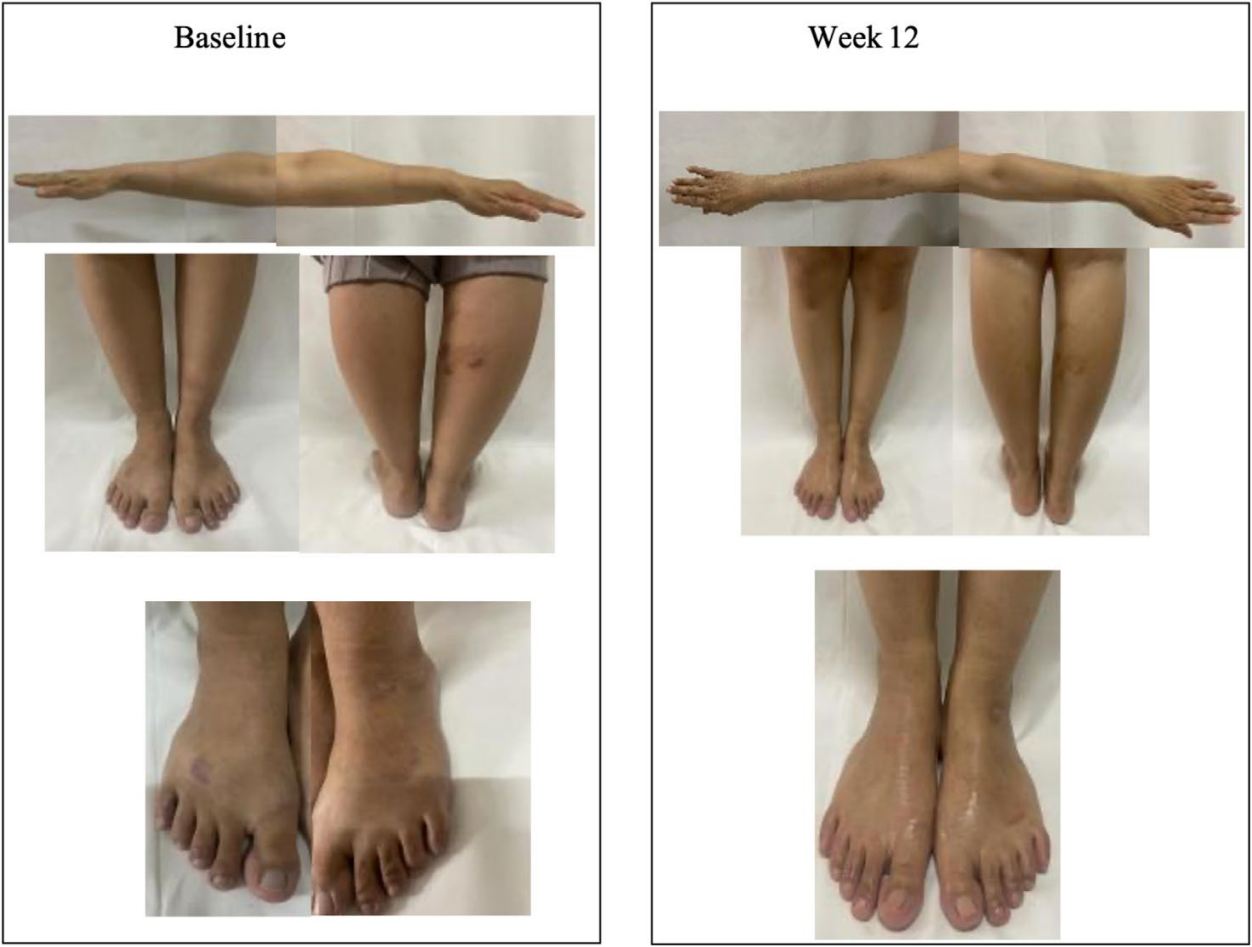
Clinical progress of subjects from Group A (emollient “plus”) from Baseline to Week 12.

**FIGURE 2:**
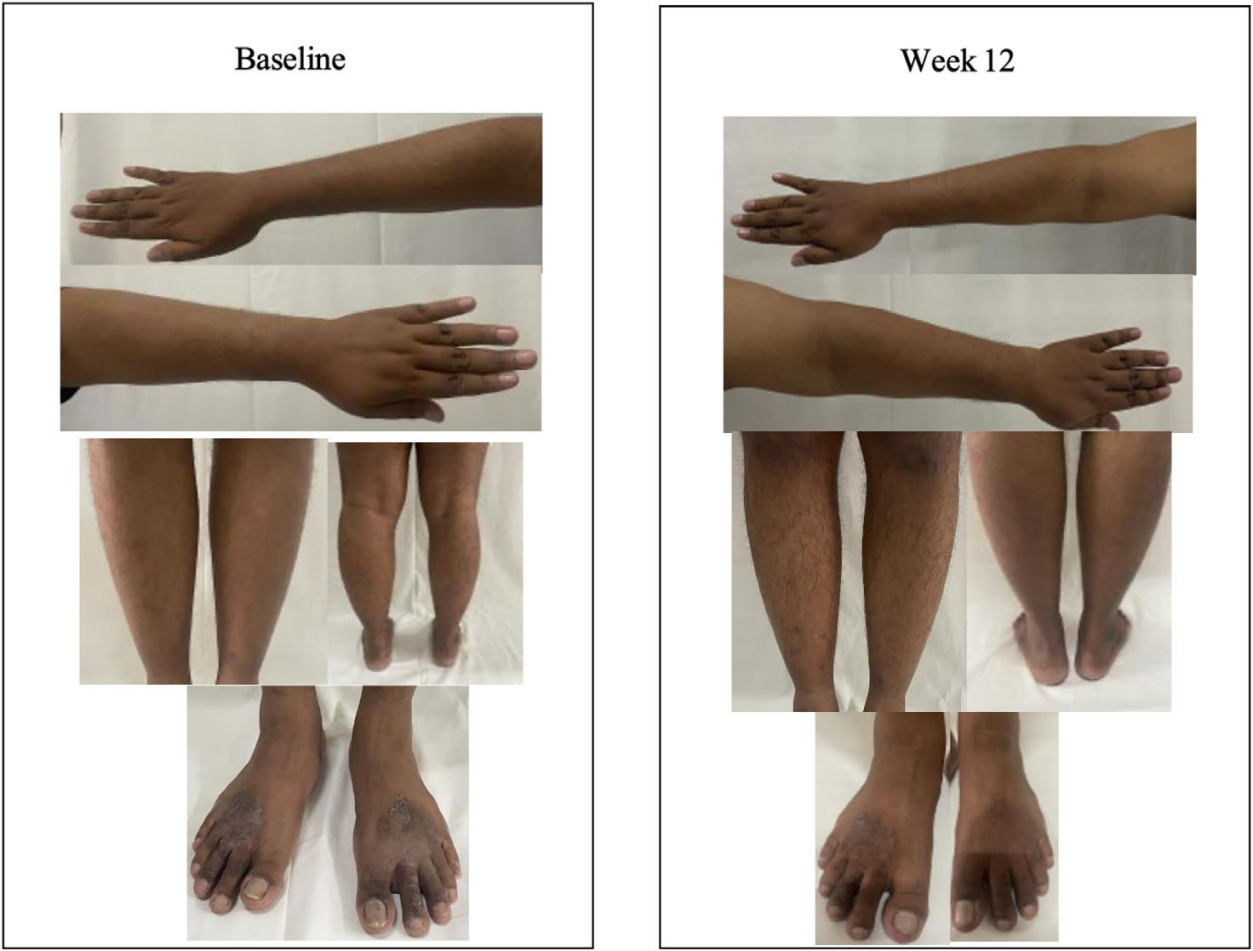
Clinical progress of subjects from Group B (urea 10%) from Baseline to Week 12.

The clinical progress observed in subjects from Baseline to Week 12 for each treatment is illustrated in Figure 1 (Group A) and Figure 2 (Group B).

There were no statistically significant differences between the groups for any of the investigated parameters at Baseline. Overall, as detailed hereafter, statistically significant differences in favor of treatment in Group A for the investigated clinical and instrumental parameters were observed throughout the study, that is, as of Week 4 (TEWL, skin pH), as of Week 8 (SCORAD, skin hydration), and at the end of treatment (Week 12: PVAS, EASI, DLQI). Of note, although less efficient than the emollient “plus,” urea 10% still provided a significant improvement of the investigated parameters.

### 3.1 Clinical Parameters

**FIGURE 3:**
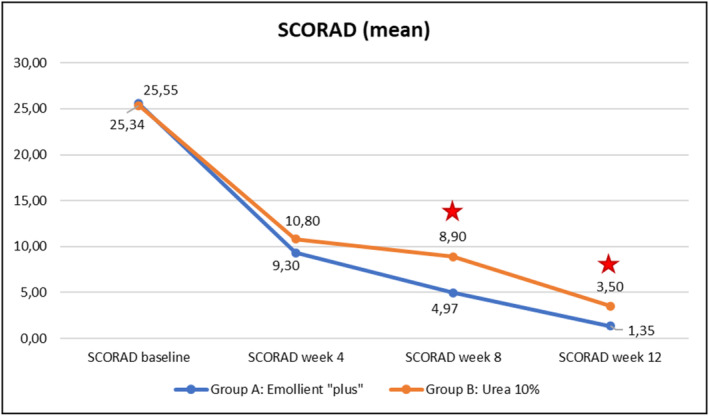
Evolution of SCORAD values over time in Groups A and B. 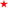 Significant difference: *p* ≤ 0.05, 95% CI.

**FIGURE 4:**
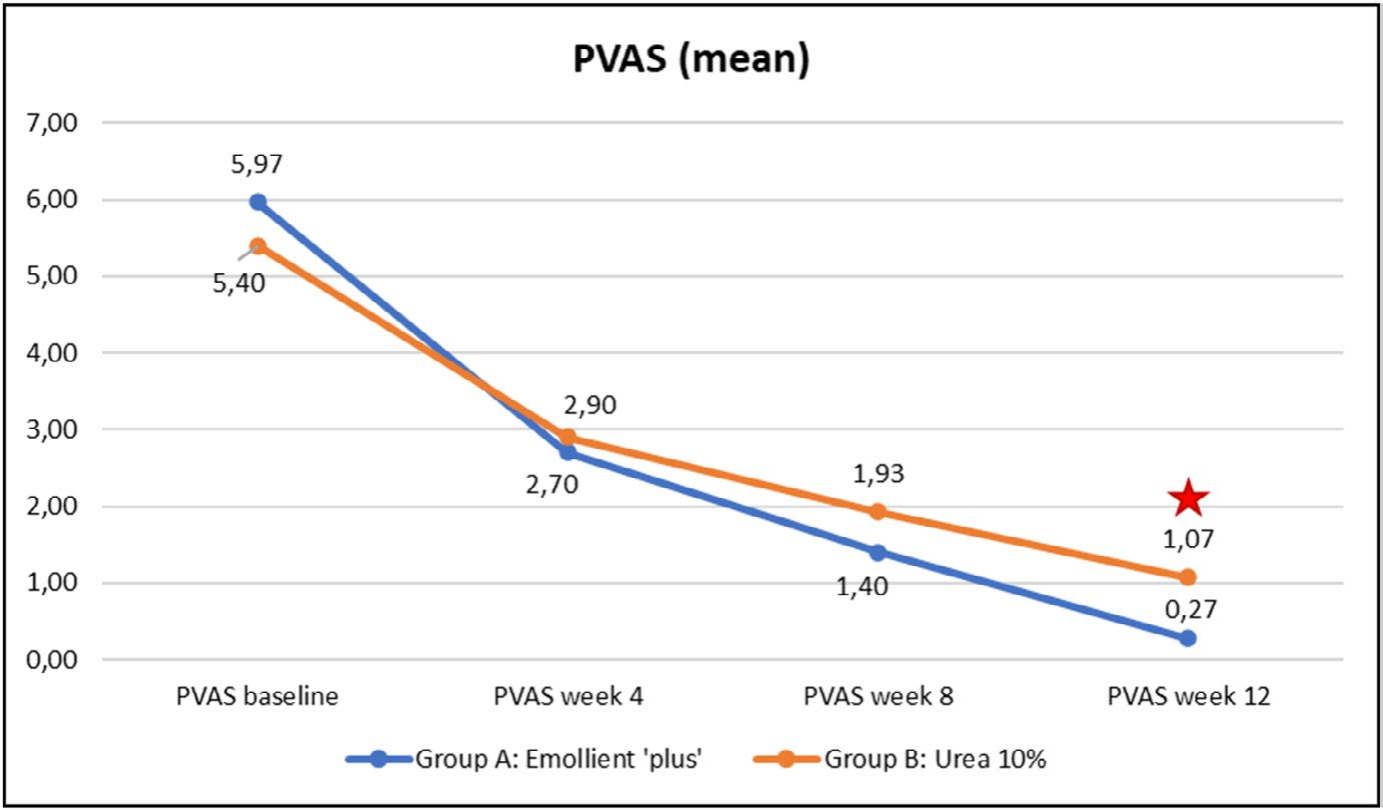
Evolution of PVAS values over time in Groups A and B. 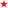 Significant difference: *p* ≤ 0.05, 95% CI.

**FIGURE 5:**
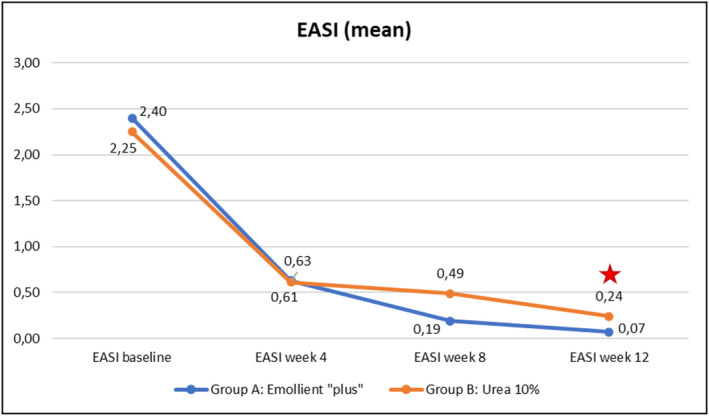
Evolution of EASI values over time in Groups A and B. 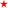 Significant difference: *p* ≤ 0.05, 95% CI.

**FIGURE 6:**
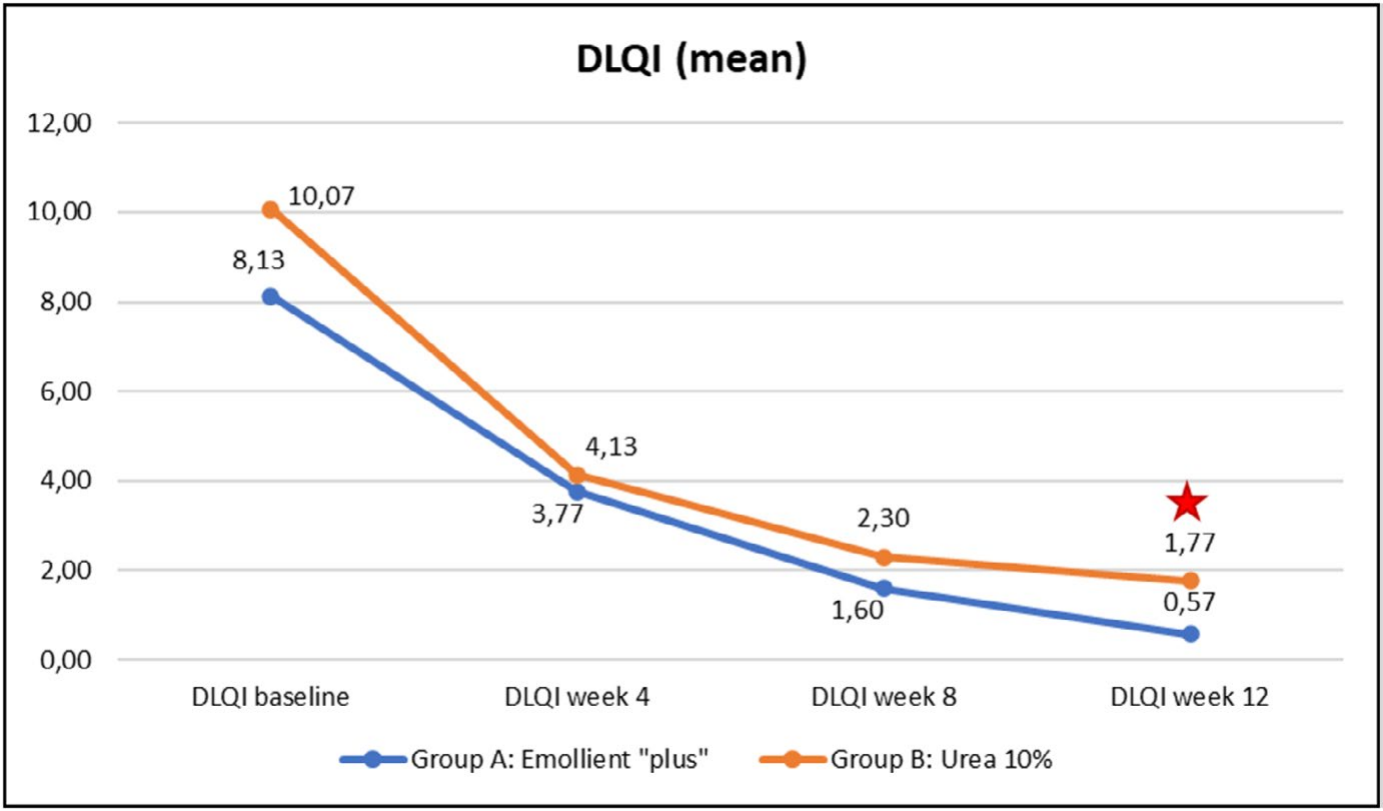
Evolution of DLQI values over time in Groups A and B. 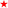 Significant difference: *p* ≤ 0.05, 95% CI.

Evolution over time of the four clinical parameters (SCORAD, PVAS, EASI, DLQI) within each of the Groups A (emollient “plus”) and B (urea 10%) is illustrated in Figures 3, 4, 5, 6 hereafter. Statistical analyses (*p*‐values) of inter‐group differences over time are presented in Table 2.

**TABLE 2:** Statistical analysis of inter‐group comparisons of clinical parameter values in favor of Group A (emollient “plus”) over time.

Evaluation time	*p* ^a^			
	SCORAD	PVAS	EASI	DLQI
Baseline	0.910	0.130	0.700	0.110
Week 4	0.360	0.470	0.560	0.840
Week 8	0.010	0.180	0.360	0.210
Week 12	0.000	0.001	0.001	0.001

^a^ Statistically significant difference: *p* ≤ 0.05, 95% CI.

These inter‐group comparisons showed that there was a statistically significant difference (*p* ≤ 0.05) for the clinical parameters in favor of the emollient “plus” at the following time points:

**Table:** 

–SCORAD (Figure 3)	Week 8 and Week 12
–PVAS (Figure 4)	Week 12
–EASI (Figure 5)	Week 12
–DLQI (Figure 6)	Week 12

### 3.2 Instrumental Parameters

**FIGURE 7:**
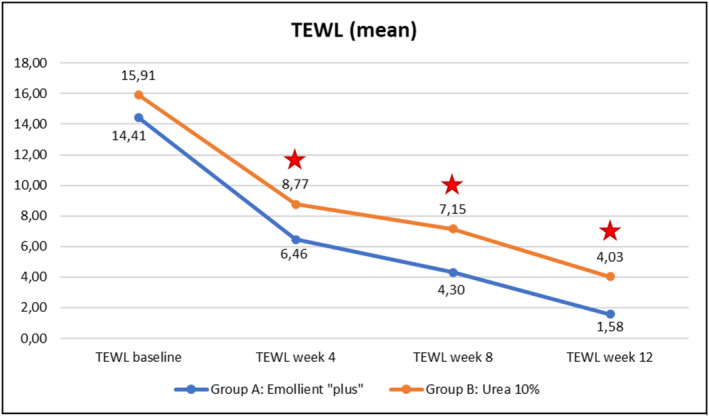
Evolution of TEWL values over time in Groups A and B. 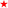 Significant difference: *p* ≤ 0.05, 95% CI.

**FIGURE 8:**
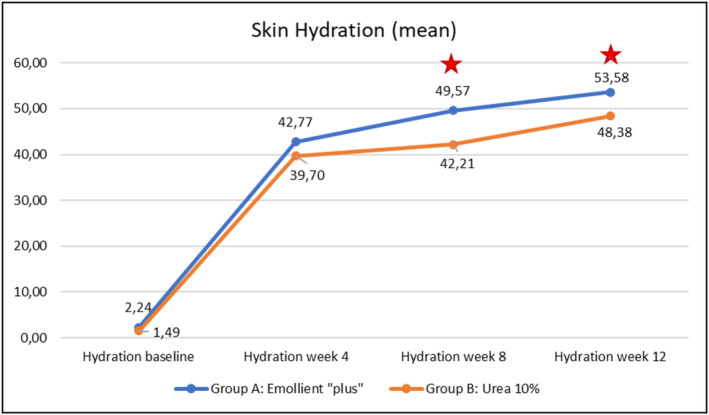
Evolution of skin hydration values over time in Groups A and B. 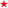 Significant difference: *p* ≤ 0.05, 95% CI.

**FIGURE 9:**
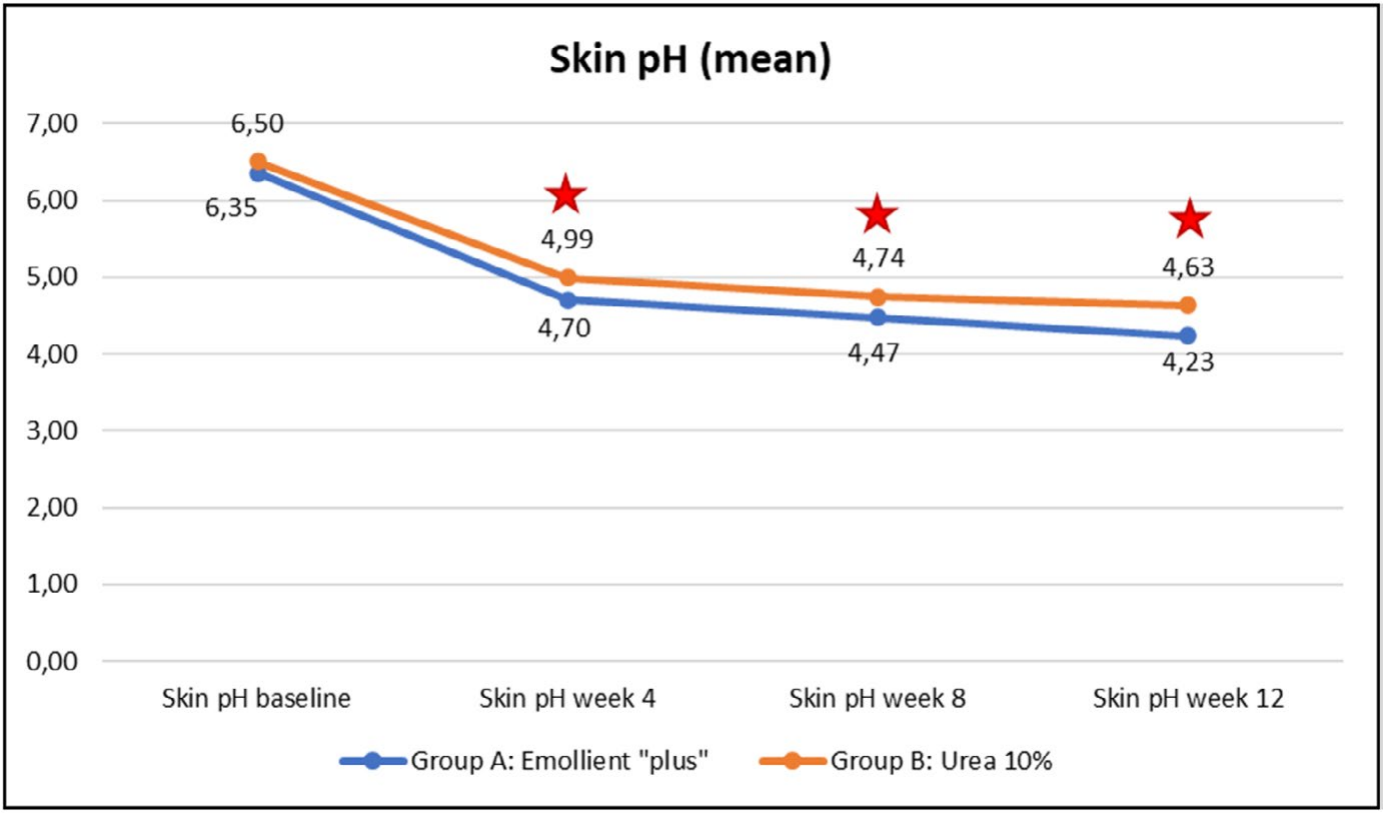
Evolution of skin pH values over time in Groups A and B. 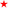 Significant difference: *p* ≤ 0.05, 95% CI.

Evolution over time of the three instrumental parameters (TEWL, skin hydration, skin pH) within each of the Groups A (emollient “plus”) and B (Urea 10%) is illustrated in Figures 7, 8, 9 hereafter. Statistical analyses (p‐values) of inter‐group differences over time are presented in Table 3.

**TABLE 3:** Statistical analysis of inter‐group comparisons of instrumental parameter values in favor of Group A (emollient “plus”) over time.

Evaluation time	*p* ^a^		
	TEWL	Skin hydration	Skin pH
Baseline	0.710	0.710	0.480
Week 4	0.050	0.360	0.030
Week 8	0.010	0.050	0.001
Week 12	0.001	0.040	0.001

^a^ Statistically significant difference: *p* ≤ 0.05, 95% CI.

These inter‐group comparisons showed that there was a statistically significant difference (*p* ≤ 0.05) for the instrumental parameters in favor of the emollient “plus” at the following time points:

**Table:** 

–TEWL (Figure 7)	Week 4, Week 8, and Week 12
–Skin hydration (Figure 8)	Week 8, Week 12
–Skin pH (Figure 9)	Week 4, Week 8, and Week 12

### 3.3 Tolerance Evaluation

Three cases of asymptomatic minimal erythema were reported in 3 of the 30 subjects (10.0%) from Group A. This event lasted for 3 days during the first week of application of the emollient “plus” and resolved without any treatment.

In Group B, 8 of the 30 subjects (26.7%) presented erythematous macules, pruritus, and xerosis, which lasted for 1 week of application of urea 10%. All patients received antihistamine therapy for 1 week (Cetirizine once daily at night) to resolve the issue.

## 4 Discussion

Atopic dermatitis is a chronic inflammatory skin disease characterized by xerosis, eczematous lesions, and pruritus with different patterns of flares and remission periods. The pathogenesis of AD is complex combining immune dysregulation and impaired skin barrier function including a microbiome disbalance with decreased microbial diversity at the favor of increased *S. aureus* , in genetically predisposed people, thus requiring a comprehensive therapeutic strategy. Moisturizers play a fundamental role in the management of AD by restoring the compromised epidermal barrier and increasing skin hydration. Simultaneously, emerging evidence suggests the crucial role of microbiome dysbiosis in AD pathophysiology, implicating a potential role of pre‐, post‐, and probiotics in restoring a diverse microbiome balance ultimately modulating skin inflammation [4]. Recent developments have allowed to include pre‐ and post‐biotics in moisturizers providing benefits in balancing the skin microbiome and immune response. Probiotics consist of live microorganisms. Pre‐biotics are components promoting the growth of beneficial microorganisms, and post‐biotics are preparations of inanimate microorganisms and/or their components that confer a health benefit on the host. Probiotics, pre‐biotics, and post‐biotics can provide immunomodulatory effects and improve skin barrier integrity through various mechanisms, including the reduction of pathogenic bacterial overgrowth and the modulation of immune system pathways, the restoration of microbial homeostasis, and the strengthening of skin defense mechanisms. Of note, pre‐biotics have the potential to reduce disease severity in AD [10]. Emollient “plus” contain active ingredients such as bacterial lysate (APF, a Vf lysate grown in thermal water, in the case of this study) considered as pre‐biotic able to improve AD symptoms and the skin microbiome of AD patients [6, 11, 12]. They can minimize the use of topical corticosteroids and topical calcineurin inhibitors, thereby reducing the side effects that arise in long‐term use [4, 5]. The key active ingredients with their mode of action of the emollient “plus” used in this study are as follows:

*Aqua posae filiformis* (Vf lysate grown in thermal water): *Vitreoscilla filiformis* is a nonpathogenic bacterium that belongs to the order Beggiatoales. This microorganism is found in hot sulfur springs. *Vitreoscilla filiformis* can stimulate the production of β‐defensins and other innate immune defense mechanisms through activation of toll‐like receptor‐2 on the surface of dendritic cells which then stimulates these cells to produce immunosuppressive interleukin‐10 (IL‐10). Release of IL‐10 results in a very high IL‐10/IL‐12 ratio for optimal T‐helper (Th) 1 or Th 2 balance and induction of T‐regulatory cell function. This will reduce pro‐inflammatory cytokines and reduce inflammation in the skin [13]. The use of Vf alone or APF (Vf lysate grown in thermal water) in an emollient “plus” formula has been shown to rebalance the skin microbiota by increasing the growth of one of the commensal bacteria, *Staphylococcus epidermidis* , and reducing *S. aureus* colonization [10, 12, 13].

Microresyl, which is extracted from *Ophiopogon japonicum* tuberous roots, has been demonstrated to decrease *S. aureus* adhesion to the skin and decrease inflammatory markers on skin cells, such as thymic stromal lymphopoietin and IL‐8 [8].

*Aqua posae filiformis* and Microresyl, key ingredients of the emollient “plus” used in Group A, can improve skin barrier function by restoring Natural Moisturizing Factors (NMF) through the reduction of the number of ceramidase‐producing bacteria, *S. aureus* , thereby increasing the amount of ceramide in the skin. Ceramide is one of the NMF ingredients which plays a role in maintaining skin hydration, skin integrity, and skin barrier function. The shea butter and niacinamide are additional ingredients with anti‐inflammatory effects further increasing ceramide production so that there is increased skin hydration which can then improve the skin barrier function [5, 13, 14].

Prior studies have shown the efficacy of the studied emollient “plus” with no issue in relation with induced contact dermatitis [8]. In the present study, no patients experienced contact dermatitis following the use of the emollient “plus.”

Benefits provided by the emollient “plus” in patients with mild‐to‐moderate AD were greater than those obtained with urea 10% moisturizer: improvements were significantly superior in Group A compared to Group B over time. Overall, the data demonstrated statistically significant differences in favor of treatment in Group A as early as Week 4 (TEWL—see Figure 7, skin pH—see Figure 9), as of Week 8 (SCORAD—see Figure 3, skin hydration—see Figure 8), and at the end of treatment (Week 12: PVAS—see Figure 4, EASI—see Figure 5, DLQI—see Figure 6). However, it should be noted that the 10% urea moisturizer used in Group B still provided good clinical results.

Urea 10% used in Group B can improve skin barrier by attracting water from the dermis or from the environment, so it can increase skin hydration and prevent moisture loss. Urea also helps improve skin barrier function by increasing antimicrobial peptides. In addition, urea regulates epidermal proliferation through increased filaggrin production without any anti‐inflammatory or anti‐pruritic effects [14].

## 5 Conclusion

In conclusion, modern emollients “plus” have proven to improve skin barrier function and rebalance microbiome, thus improving clinical signs and symptoms of patients with AD, either used alone in the milder forms or in adjunct to topical or systemic drugs for the more severe types [8]. Indeed, emollients and emollients “plus” are recommended by European guidelines on AD [6]. Previous studies on emollient “plus” have shown superior efficacy compared to a regular emollient on AD and on maintaining a diverse microbiome [12]. In the present study, results obtained in the AD subjects of Groups A and B showed continuous improvements in all the investigated clinical and instrumental parameters throughout the 12‐week treatment, beneficial effects being observed as early as at Week 4 of treatment. Of note, the emollient “plus” showed a greater improvement on disease severity and on skin hydration, with a better tolerance compared to the local standard of emollient care, that is, urea 10%.

In terms of tolerance, one of the known adverse effects of moisturizers is skin irritation, especially on excoriated AD skin. In a comparative single‐blind randomized study of the potential for irritation of a single application of commercially available moisturizers in AD children aged between 8 and 16 years, a ceramide‐based cream had a significantly lower irritant score than a urea 5% cream [15]. There were no major tolerance issues in the present study. Temporary (3 days) minimal erythema was reported for three subjects treated with the emollient “plus” and did not require any treatment for clearing. Eight subjects of the urea 10% group experienced erythematous macules, pruritus, and xerosis which disappeared after 1 week of treatment with oral anti‐histamine.

The limitations of this study were the lack of objective measurement of skin inflammation and other clinical parameters such as elasticity. Regarding inflammation, especially erythema, this could be objectively assessed using a mexameter. Of other clinical parameters, skin elasticity assesses the state of structurally hydrated skin. Therefore, if a comparable study is conducted in the future with an extended treatment time, skin elasticity should be considered as a noteworthy parameter for evaluation. Skin barrier defects in patients with AD reflecting changes in the chemical composition of the *stratum corneum* correlating with clinical severity can also be investigated using infrared spectroscopy [16].

## Author Contributions

All authors made a significant contribution to the work reported, whether that is in the conception, study design, execution, acquisition of data, analysis, and interpretation, or in all these areas; took part in drafting, revising, or critically reviewing the article; gave final approval of the version to be published; have agreed on the journal to which the article has been submitted; and agree to be accountable for all aspects of the work.

## Ethics Statement

The procedures followed were in accordance with the ethical standards of the responsible committee on human experimentation in Dr. Soetomo General Academic Hospital, Surabaya, Indonesia (0672/KEPK/5/2023), and with the Helsinki Declaration of 1975, as revised in 1983.

## Consent

The patients in this manuscript have given written informed consent to publication of their case details including photographs.

## Conflicts of Interest

Delphine Kerob is an employee of La Roche‐Posay Laboratoire Dermatologique. The other authors declare no conflicts of interest.

## Data Availability

The data that support the findings of this study are available from the corresponding author upon reasonable request.

## References

[1] H. H. Kong , J. Oh , C. Deming , et al., “Temporal Shift in the Skin Microbiome Associated With Disease Flares and Treatment in Children With Atopic Dermatitis,” Genome Research 22, no. 5 (2012): 850–859. 22310478 10.1101/gr.131029.111 PMC3337431

[2] M. Hammond , A. Gamal , P. K. Mukherjee , et al., “Cutaneous Dysbiosis May Amplify Barrier Dysfunction in Patients With Atopic Dermatitis,” Frontiers in Microbiology 13 (2022): 944365, 10.3389/fmicb.2022.944365. 36452925 PMC9701744

[3] I. Garcia‐Jiménez , L. Sans‐de San Nicolàs , L. Curto‐Barredo , et al., “Heterogeneous IL‐9 Production by Circulating Skin‐Tropic and Extracutaneous Memory T Cells in Atopic Dermatitis Patients,” International Journal of Molecular Sciences 25 (2024): 8569, 10.3390/ijms25168569. 39201262 PMC11354683

[4] M. Trzeciak , W. Zysk , and D. Wolańska‐Buzalska , “Emollients ‘Plus’ With *Vitreoscilla filiformis* in Monotherapy and Adjunctive Therapy in Skin Diseases in Children,” Dermatology Review 110 (2023): 602–607.

[5] H. Zelenkova , D. Kerob , S. Salah , and A. Demessant‐Flavigny , “Impact of Daily Use of Emollient ‘Plus’ on Corticosteroid Consumption in Patients With Atopic Dermatitis: An Open, Randomized Controlled Study,” Journal of the European Academy of Dermatology and Venereology 37 (2023): 27–34. 10.1111/jdv.18947 37092256

[6] A. Wollenberg , S. Barbarot , T. Bieber , et al., “Consensus‐Based European Guidelines for Treatment of Atopic Eczema (Atopic Dermatitis) in Adults and Children: Part I,” Journal of the European Academy of Dermatology and Venereology 32 (2018): 657–682, 10.1111/jdv.14891. 29676534

[7] Y. F. Mahe , M. J. Perez , C. Tacheau , et al., “New *Vitreoscilla filiformis* Extract Grown on Spa Water‐Enriched Medium Activates Endogenous Cutaneous Antioxidant and Antimicrobial Defenses Through a Potential Toll‐Like Receptor 2/Protein Kinase C, Zeta Transduction Pathway,” Clinical, Cosmetic and Investigational Dermatology 6 (2013): 191–196, 10.2147/CCID.S47324. 24039440 PMC3770492

[8] C. C. Ch'ng , “Rebalancing of the Skin Microbiome With an Emollient ‘Plus’ for Effective Management of Atopic Dermatitis: A Mini Review,” Medical Journal of Malaysia 79, no. 2 (2024): 203–205. 38553927

[9] C. Correa and J. Nebus , “Management of Patients With Atopic Dermatitis: The Role of Emollient Therapy,” Dermatology Research and Practice 2012 (2012): 836931, 10.1155/2012/836931. 23008699 PMC3449106

[10] A. Gueniche , B. Knaudt , E. Schuck , et al., “Effects of Nonpathogenic Gram‐Negative Bacterium *Vitreoscilla filiformis* Lysate on Atopic Dermatitis: A Prospective, Randomized, Double‐Blind, Placebo‐Controlled Clinical Study,” British Journal of Dermatology 159, no. 6 (2008): 1357–1363, 10.1111/j.1365-2133.2008.08836.x. 18795916

[11] A. Wollenberg , M. Kinberger , B. Arents , et al., “First Update of the Living European Guideline (EuroGuiDerm) on Atopic Eczema,” Journal of the European Academy of Dermatology and Venereology 37, no. 11 (2023): e1283–e1287, 10.1111/jdv.19269. 37328919

[12] S. Seité , H. Zelenkova , and R. Martin , “Clinical Efficacy of Emollients in Atopic Dermatitis Patients—Relationship With the Skin Microbiota Modification,” Clinical, Cosmetic and Investigational Dermatology 10 (2017): 25–33, 10.2147/CCID.S121910. 28138262 PMC5238811

[13] N. Magnolo , T. Jaenicke , A. Tsianakas , et al., “Comparison of Different Skin Care Regimens in Patients With Moderate to Severe Atopic Dermatitis Receiving Systematic Treatment: A Randomized Controlled Trial,” Journal of the European Academy of Dermatology and Venereology 37, no. 5 (2023): 18–26. 10.1111/jdv.18949 37092275

[14] C. Messaraa , J. Drevet , D. Jameson , G. Zuanazzi , and I. De Ponti , “Can Performance and Gentleness Be Reconciled? A Skin Care Approach for Sensitive Skin,” Cosmetics 9, no. 2 (2022): 34, 10.3390/cosmetics9020034.

[15] V. P. Y. Ho , E. Ma , H. M. Liew , M. S. Y. Ng , and M. J. A. Koh , “Comparing the Potential for Irritation of a Ceramide‐Based Moisturizer With a Urea‐Based Moisturizer for Pediatric Atopic Dermatitis,” Dermatology and Therapy 10 (2020): 807–813, 10.1007/s13555-020-00388-6. 32372387 PMC7367988

[16] S. F. Williams , H. Wan , J. Chittock , et al., “Characterization of Skin Barrier Defects Using Infrared Spectroscopy in Patients With Atopic Dermatitis,” Clinical and Experimental Dermatology 49 (2024): 466–477, 10.1093/ced/llad416. 38011533

